# Functional characterisation of a nicotinic acetylcholine receptor α subunit from the brown dog tick, *Rhipicephalus sanguineus*^☆^

**DOI:** 10.1016/j.ijpara.2013.11.002

**Published:** 2014-01

**Authors:** Kristin Lees, Andrew K. Jones, Kazuhiko Matsuda, Miki Akamatsu, David B. Sattelle, Debra J. Woods, Alan S. Bowman

**Affiliations:** a Institute of Biological and Environmental Sciences, University of Aberdeen, Tillydrone Ave, Aberdeen AB24 2TZ, UK; b Faculty of Life Sciences, University of Manchester, Oxford Rd, Manchester M13 9PT, UK; c Department of Biological and Medical Sciences, Faculty of Health and Life Sciences, Oxford Brookes University, Oxford OX3 0BP, UK; d Department of Applied Biological Chemistry, Faculty of Agriculture, Kinki University, 3327-204 Nakamachi, Nara 631-8505, Japan; e Wolfson Institute for Biomedical Research, Cruciform Building, University College London, Gower Street, London WC1E 6BT; f Pfizer Animal Health, Pfizer Ltd, Sandwich, Kent CT13 9NJ, UK

**Keywords:** *Rhipicephalus sanguineus*, Tick, Ion channel, Acaricide, Nicotinic acetylcholine receptor, *Xenopus* oocytes, Imidacloprid

## Abstract

Ticks and tick-borne diseases have a major impact on human and animal health worldwide. Current control strategies rely heavily on the use of chemical acaricides, most of which target the CNS and with increasing resistance, new drugs are urgently needed. Nicotinic acetylcholine receptors (nAChRs) are targets of highly successful insecticides. We isolated a full-length nAChR α subunit from a normalised cDNA library from the synganglion (brain) of the brown dog tick, *Rhipicephalus sanguineus*. Phylogenetic analysis has shown this *R. sanguineus* nAChR to be most similar to the insect α1 nAChR group and has been named Rsanα1. Rsanα1 is distributed in multiple tick tissues and is present across all life-stages. When expressed in *Xenopus laevis* oocytes Rsanα1 failed to function as a homomer, with and without the addition of either *Caenorhabditis elegans* resistance-to-cholinesterase (*RIC*)-3 or *X. laevis RIC*-3. When co-expressed with chicken β2 nAChR, Rsanα1 evoked concentration-dependent, inward currents in response to acetylcholine (ACh) and showed sensitivity to nicotine (100 μM) and choline (100 μM). Rsanα1/β2 was insensitive to both imidacloprid (100 μM) and spinosad (100 μM). The unreliable expression of Rsanα1 in vitro suggests that additional subunits or chaperone proteins may be required for more robust expression. This study enhances our understanding of nAChRs in arachnids and may provide a basis for further studies on the interaction of compounds with the tick nAChR as part of a discovery process for novel acaricides.

## Highlights

• A nicotinic acetylcholine receptor α-subunit (Rsanα1) was identified in *Rhipicephalus sanguineus*.
• Rsanα1 was not restricted to the synganglion (“brain”).
• Rsanα1 was functionally expressed in *Xenopus* oocytes.
• Rsanα1 responded to acetylcholine, nicotine and choline.
• Rsanα1 was unresponsive to imidacloprid and spinosad.

## Graphical abstract

**Figure:**
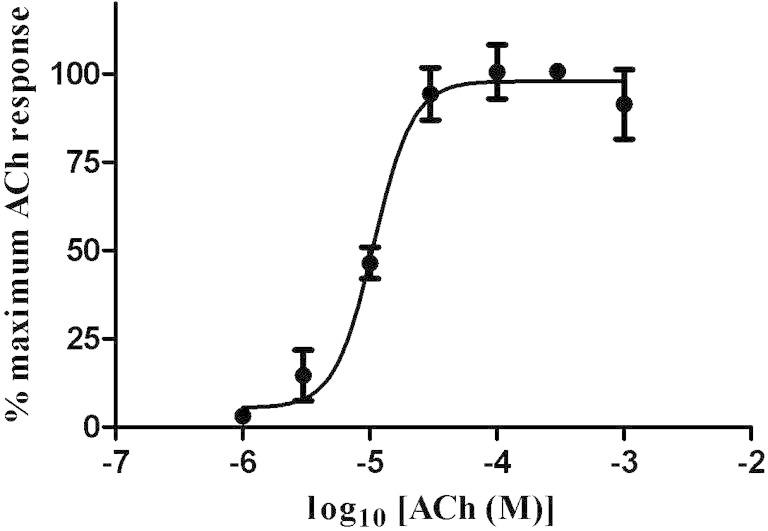

## 1 Introduction

Ticks are by far the most economically important ectoparasite of global livestock production and are also important vectors of human diseases (such as tick-borne encephalitis, Lyme borreliosis) and animal diseases (including babesiosis and theileriosis) (Jongejan and Uilenberg, 2004). Within the animal health industry, anti-parasitic drug discovery costs an estimated $25 million per year (Woods and Williams, 2007) and an important component of this research includes products for ticks on companion animals (cats and dogs), in particular the brown dog tick, *Rhipicephalus sanguineus*, the most widely distributed tick species in the world (Inokuma et al., 1997; Gray et al., 2013).

The control of ticks relies heavily on chemical acaricides (Taylor, 2001; George et al., 2004). However, there is now increasing resistance to every available acaricide, necessitating the urgent development of new treatments (George et al., 2004). Most existing acaricides target the tick nervous system. However, the field of tick neurobiology lags quite considerably behind that of other arthropod groups. The cholinergic system of the tick (see Lees and Bowman, 2007) has been validated as an acaricidal target ever since tobacco extracts containing nicotine were used as an ectoparasiticide, suggesting evidence for the presence of nicotinic acetylcholine receptors (nAChRs) in the tick (Meinke, 2001). Recently, the spinosyn insecticides which target nAChRs (Geng et al., 2013) have been shown to be effective acaricides against ticks known to be pyrethroid- and organophosphate-resistant (Miller et al., 2013). Hence the nAChRs have been validated as viable targets for acaricide development.

The neonicotinoid insecticides, of which imidacloprid is the best known, target the insect nAChR and account for nearly 17% of the global insecticide market (Jeschke and Nauen, 2008), acting on the nAChR at a different site from the spinosyns. Although imidacloprid displays a strong efficacy against insects (Meinke, 2001), the Acari, including both ticks and mites, are largely insensitive to imidacloprid and it appears that the basis of this insensitivity may reside at the nAChR itself (Erdmanis et al., 2012). This is of particular commercial interest as the combination flea-tick treatments available for cats and dogs often contain imidacloprid which is effective against pet fleas (*Ctenocephalides felis* and *Ctenocephalides canis*) but ineffective against ticks. Thus different formulations, often including pyrethroids, are administered to enable efficacy against both the fleas and ticks.

To identify potential new acaricide targets in the acarine nervous system, a normalised cDNA library was prepared from the synganglia of unfed adult *R. sanguineus* ticks. Among the 1,000 expressed sequence tags (ESTs) sequenced (Lees et al., 2010) a full-length nAChR subunit was detected. Here, we report functional characteristics of the first identified full-length tick α nAChR subunit.

## 2 Materials and methods

### 2.1 Tick material

Eggs, larvae, nymphs and adults of the brown dog tick, *R. sanguineus*, were supplied by Charles River, Carrentrila, Ireland.

### 2.2 Cloning of Rsanα1 from *R. sanguineus*

As part of a transcriptomic analysis of the adult *R. sanguineus* synganglion, 1,008 ESTs were sequenced from a normalised, full-length cDNA library, as described in Lees et al. (2010). One clone was identified as a partial putative nAChR subunit and re-sequenced to obtain the full length of the gene.

### 2.3 Tissue and life-stage specificity of Rsanα1 expression

All experiments involving animals were carried out in compliance with national legislation and subject to local ethical review. *Rhipicephalus sanguineus* eggs (one egg mass), larvae (0.05–0.1 mg), nymphs (20–25), individual unfed whole adults and synganglia (20–25) from a mixed male/female population of unfed adults were stored in Trizol at −80 °C and homogenised using a Mixer Mill MM300 (Retsch, Haan, Germany). Total RNA was extracted using the phenol/chloroform extraction method according to the manufacturer’s protocol (Invitrogen, Paisley, UK). Adult *R. sanguineus* were fed on guinea pigs and removed on day 8 post-infestation, as described by Ball et al. (2009). Tissues including the synganglion, salivary glands, gut, Malpighian tubules and oviduct were dissected from individual fed adult female *R. sanguineus*, immediately frozen using dry ice and stored at −80 °C. Total RNA was purified from individual tick tissues using the Mini RNA Isolation Kit™ (Zymo Research, Orange, California, USA) according to the manufacturer’s instructions. cDNAs were synthesised, including DNAse treatment, as described previously (Lees et al., 2010) and concentrations were determined with an ND-100 microspectrophotometer (Nanodrop Technologies, Wilmington, Denver, USA) and adjusted to 1 μg/μl with nuclease-free water. The material and reverse-transcription (RT)-PCR procedures were validated by PCR using primers for actin, generating a fragment of approximately 110 bp, as described in Ball et al. (2009). Nested-PCR using gene-specific primers based on the nAChR identified in the *R. sanguineus* synganglion library by EST screening (Lees et al., 2010) was used to detect the presence of transcripts in tissue. For round one, the primers used were: F1 5′ cgttcgacaagcaggact 3′ and R1 5′ tcagacatctgtccagggatag3′ followed by F1 5′ cgttcgacaagcaggact 3′ and R2 5′ atcatagagcgaaggagcctgg 3′. The PCR conditions (using Bioline reagents) consisted of 35 cycles using the following: one cycle of 94 °C for 2 min, 35 cycles of 94 °C for 30 s, 58 °C for 1 min and 72 °C for 1 min 15 s, with 5 min at 72 °C to finish. The first round template (1 μl) was used as template for the second round of PCR using the conditions described above for 30 cycles.

### 2.4 Expression in *Xenopus* oocytes

The *R. sanguineus* α1 (Rsanα1) nAChR was subcloned into the expression vector pcDNA 3.1 V5/His-TOPO-TA (Invitrogen) following the manufacturer’s instructions. cDNAs encoding chicken β2 nAChR, *Caenorhabditis elegans RIC-3* (*Cele-RIC-3*) and *Xenopus laevis RIC-3* (Xla-*RIC-*3) were provided by Professor Marc Balllivet (University of Geneva, Switzerland), Professor Millet Treinin (The Hebrew University of Jerusalem, Israel) and Professor Adrian Wolstenholme (University of Georgia, USA) respectively. Plasmids were linearised with *Eco*RV (Rsanα1), *Xba*I (chick β2), *Not*I (Cele-*RIC*-3) and *Nco*I (Xla-*RIC*-3), respectively. cRNAs were generated using the T7 or SP6 mMessage Machine Kits (Ambion Ltd, Warrington, UK).

Oocytes at stages V or VI of development were obtained from mature female *X. laevis* frogs and were treated with 2 mg/ml of collagenase (Sigma–Aldrich, Poole, UK) for 1 h in a calcium-free version of standard oocyte saline (SOS). The composition of SOS was as follows: 100 mM NaCl, 2 mM KCl, 1.8 mM CaCl_2_, 1 mM MgCl_2_, 5 mM HEPES at pH 7.6. Following collagenase treatment, the follicular layer was removed manually using fine forceps. Fifty nl of cRNA (1 μg/μl) of Rsanα1 alone, Rsanα1/Cele-*RIC*-3 (1:0.2), Rsanα1/Xla-*RIC*-3 (1:0.2), Rsanα1/chick β2 (1:1), Rsanα1/chick β2/Cele-*RIC*-3 (1:1:0.2) or Rsanα1/chick β2/Xla-*RIC*-3 (1:1:0.2) were injected into the cytoplasm. Following injections, oocytes were incubated at 18 °C in SOS supplemented with 2.5 mM sodium pyruvate, 100 U/ml of penicillin, 100 μg/ml of streptomycin and 50 μg/ml of gentamicin for 3–4 days.

### 2.5 Electrophysiology

Oocytes were secured in a Perspex chamber which was perfused continuously with SOS at a constant flow rate (4 ml min^−1^) via a gravity-fed system (Buckingham et al., 1994). Atropine (0.5 μM) was included in the saline to suppress responses from endogenous muscarinic AChRs (Lupu-Meiri et al., 1990; Blake et al., 1993). Membrane currents were measured using the two-electrode voltage clamp method (as described in Buckingham et al., 2006) where the oocyte membrane was clamped at −100 mV. Nicotine, ACh, choline, imidacloprid and spinosad (all Sigma–Aldrich) were diluted with SOS containing 0.5 μM atropine immediately prior to experimentation. Oocytes were challenged for 5 s with increasing concentrations of ACh at intervals of 3–5 min to minimise the effects of desensitisation. The maximum amplitude of the current recorded for each challenge was normalised to the response to 300 μM ACh. Using GraphPad Prism version 4.0 (GraphPad Software Inc. USA), normalised data were fitted to the following equation: *Y* = *I*_min_ + (*I*_max_ − *I*_min_)/1 + 10(^logEC^_50_^−^*^X^*)*^n^*_H_ where *Y* is the normalised response amplitude to a compound applied at concentration *X*, *I*_max_ and *I*_min_ are the maximum and minimum normalised responses respectively. EC_50_ is the concentration giving half the maximum normalised response and *n*_H_ is the Hill co-efficient (Hosie and Sattelle, 1996).

## 3 Results

### 3.1 Rsanα1, a cys-loop family, nAChR α subunit

**Fig. 1:**
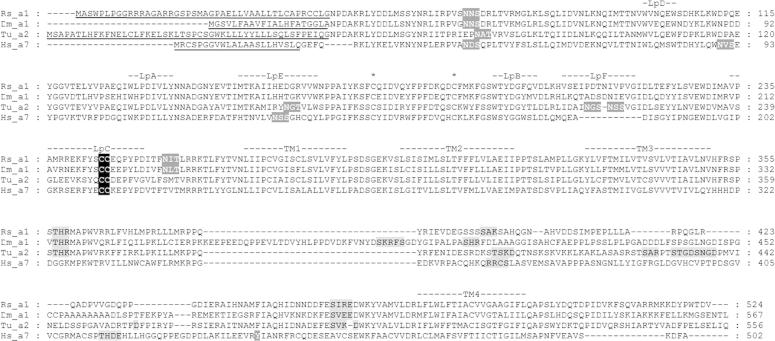
Protein sequence alignment of the *Rhipicephalus sanguineus* α1 (Rsanα1) nicotinic acetylcholine receptor (nAChR) subunit. *Drosophilia melanogaster* (Dm) α1, *Tetranychus urticae* (Tu) α2 and *Homo sapiens* (Hs) α7 nAChRs are included for comparison. The N-terminal signal peptides are underlined. The positions of loops (LpA-F) which are implicated in ligand binding and the four transmembrane domains (TM1–4) are indicated. The sites of cysteine residues involved in the cys-loop are marked with asterisks and the vicinal cysteine residues characteristic of α-type nAChR are highlighted in black. Potential glycosylation and putative phosphorylation sites are highlighted in grey.

A full-length nAChR (1,895 bp) subunit was identified containing an open reading frame coding for 525 amino acids and a 3′ untranslated region (UTR) of 317 bp with a poly A tail. This sequence possesses features typical of nAChR subunits including an N-terminal signal peptide, an extracellular N-terminal domain with loops A–F which are involved in ligand binding, a cysteine loop which consists of two disulphide bond-forming cysteines separated by 13 amino acid residues, four transmembrane domains (TM1–TM4) and a large, highly variable intracellular loop between TM3 and TM4 (Fig. 1). The presence of two adjacent cysteines in loop C signifies that this is an α-subunit (Kao et al., 1984). This sequence has been deposited in GenBank (accession number **KF695387**).

**Fig. 2:**
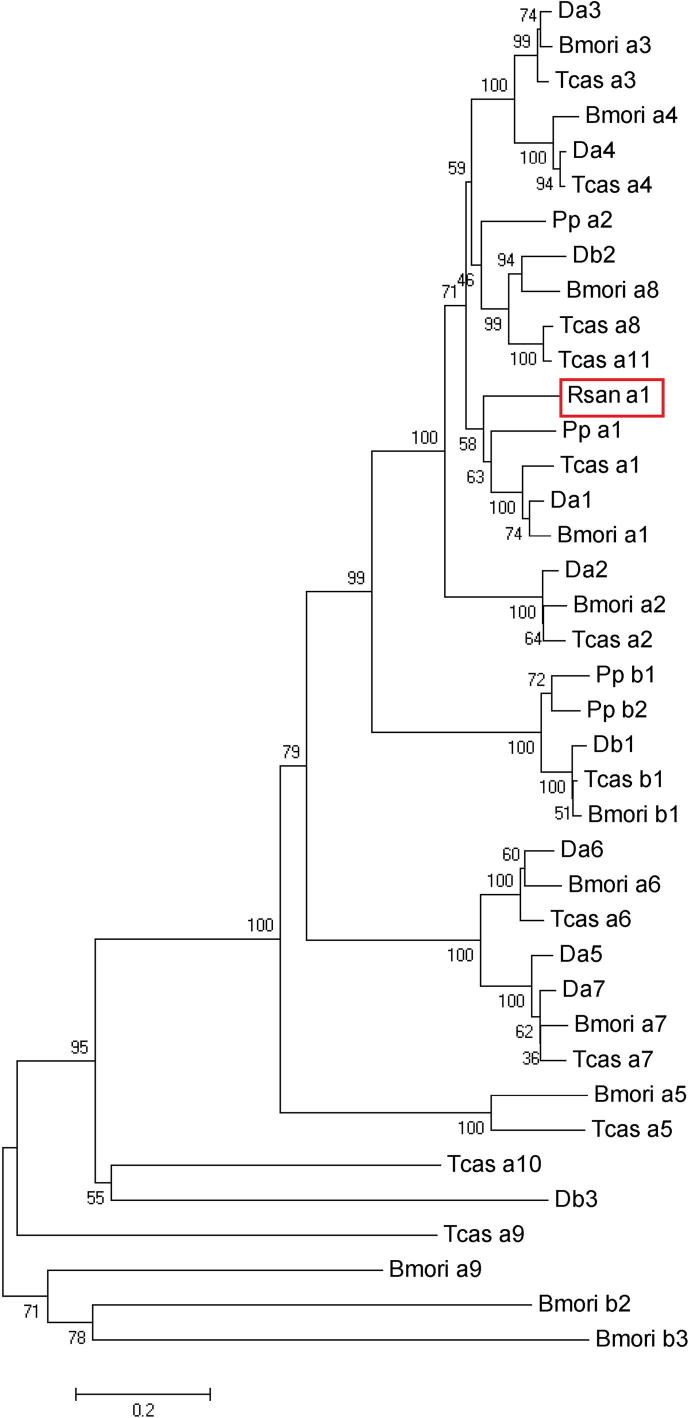
Phylogenetic analysis of the *Rhipicephalus sanguineus* α nicotinic acetylcholine receptor (nAChR) subunit. Unrooted phylogenetic tree containing the identified *R. sanguineus* nAChR subunit. Sequences were aligned using ClustalX2. The phylogenetic tree was constructed using protein sequences with the Neighbor-Joining (NJ) method using MEGA version 5 (Kumar et al., 2008). Scale bar represents an estimate of the number of amino acid substitutions per site and numbers represent bootstrap values with 1,000 replications. Accession and database sequence identifiers are as follows: *Drosophila melanogaster*: Dα1 (X07194), Dα2(X53583), Dα3 (CAA75688), Dα4, (AJ272159); Dα5 (AF272778), Dα6 (AF321445), Dα7, (AY036614), Dβ1 (CAA27641), Dβ2, (CAA39211), Dβ3, (CAC48166). *Bombyx mori*: Bmoriα1, (EU082074); Bmoriα2, (EU082075); Bmoriα3 (EU082076), Bmoriα4, (**EU82077**); Bmoriα5, (EU082080); Bmoriα6, (EU082082); Bmoriα7, (EU082084), Bmoriα8, (EU082085); Bmoriα9, (EU082087), Bmoriβ1 (**EU82071**), Bmoriβ2 (EU082072), Bmoriβ3 (EU082073). *Tribolium castaneum*: Tcasα1 (EF526080), Tcasα2 (EF526081), Tcasα3 (EF526082), Tcasα4 (EF526083), Tcasα5 (EF526085), Tcasα6 (EF526086), Tcasα7 (EF526089), Tcasα8 (EF526090), Tcasα9 (EF526091), Tcasα10 (EF526092), Tcasα11 (EF526093), Tcasβ1 (EF526094). *Pardosa pseudoannulata*: Ppα1 (**HM01780**) Ppα2 (ADG63462), Ppβ1 (GQ259335), Ppβ2 (ADG63463).

BLASTx analysis showed this sequence to be most similar to arthropod nAChRs with the highest hits: *Ixodes scapularis* putative β1 nAChR (**EEC04113**) (*E* = 0), a predicted α1-like nAChR from *Metaseiulus occidentalis* (XP_003745680) (*E* = 0), *Pardosa pseudoannulata* α2 nAChR (**ADG63462**) (*E* = 0) and *Bombyx mori* α1 (**ABV72683**) (*E* = 0). Phylogenetic analysis of the protein sequences with identified insect nAChR gene families from *Drosophila melanogaster*, *B. mori* and *Tribolium castaneum* indicated that this *R. sanguineus* α subunit is most similar to the Dα1 group (Fig. 2). Based on this similarity, we have termed this subunit Rsanα1 nAChR.

### 3.2 Tissue and life stage distribution

**Fig. 3:**
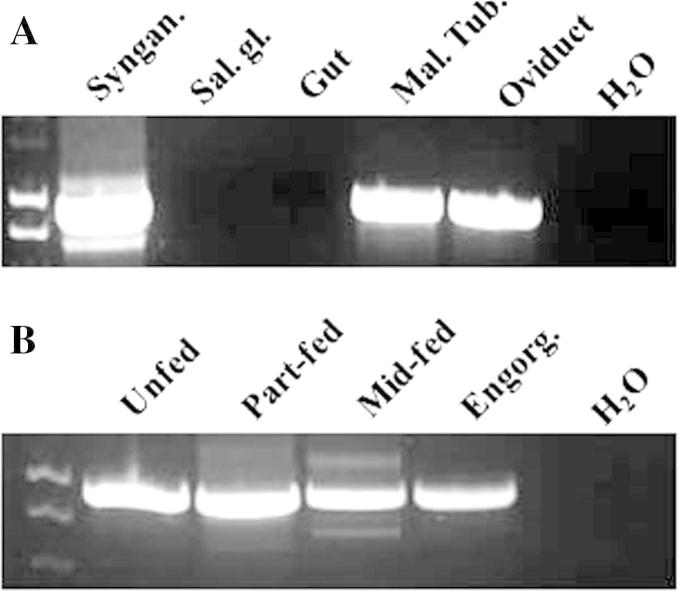
Tissue and temporal distribution of Rsanα1 nicotinic acetylcholine receptor (nAChR) in adult *Rhipicephalus sanguineus*. The quality of the cDNA was validated by PCR for actin (data not shown) and then the cDNA was adjusted to 1 μg/μl for each tissue. The products were obtained from nested-PCR using gene-specific primers. (A) Tissue expression pattern of Rsanα1 in partially fed adult female *R. sanguineus*. Lane 1, synganglion tissue; lane 2, salivary glands; lane 3, gut; lane 4, Malpighian tubules; lane 5, oviduct tissue and lane 6, negative control (water). (B) Temporal distribution of Rsanα1. Lane 1, unfed adult synganglion cDNA (mixed male and female); lane 2, partially fed individual female tick adult synganglion cDNA (tick weight = 6.3 mg); lane 3, partially-fed individual female adult tick synganglion (tick weight = 51.2 mg); lane 4, fully engorged female tick synganglion (tick weight = 132 mg) and lane 5, negative control (water).

**Fig. 4:**
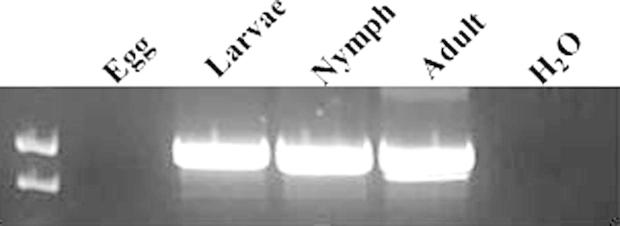
Life-stage distribution of *Rhipicephalus sanguineus Rsanα1* nicotinic acetylcholine receptor (nAChR). The quality of the cDNA was validated by PCR for actin (data not shown) and then the cDNA was adjusted to 1 μg/μl for each tissue. Nested PCR using gene-specific primers were used to detect the presence of *Rsanα1* across different life stages of *R. sanguineus*. Lane 1, eggs; lane 2, larval tissue; lane 3, nymphal tissue; lane 4, whole unfed adult cDNA and lane 5, negative control (water).

Rsanα1 transcript was detected by nested RT-PCR in the synganglion, Malpighian tubules and oviduct of partially fed *R. sanguineus* females but was absent in the salivary glands and gut tissues (Fig. 3A). In the synganglion of adult females, Rsanα1 was expressed through the feeding cycle from the unfed through to the engorged and detached phases (Fig. 3B). Rsanα1 was also detected in larvae, nymphs and unfed whole adult (mixed sex) ticks but was not detected in developing eggs (Fig. 4).

### 3.3 Functional heterologous expression of Rsanα1 in *X. laevis* oocytes

**Fig. 5:**
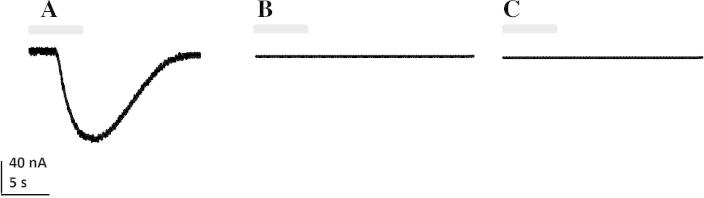
Representative two-electrode voltage clamp current traces from *Xenopus* oocytes expressing Rsanα1/β2 to (A) 1 mM acetylcholine (ACh), (B) 100 μM imidacloprid (IMI) and (C) 100 μM spinosad following a 5 s exposure (grey bar) to each compound. Only oocytes displaying responses to ACh were then challenged with the test compounds (*n* > 4 for all compounds).

**Fig. 6:**
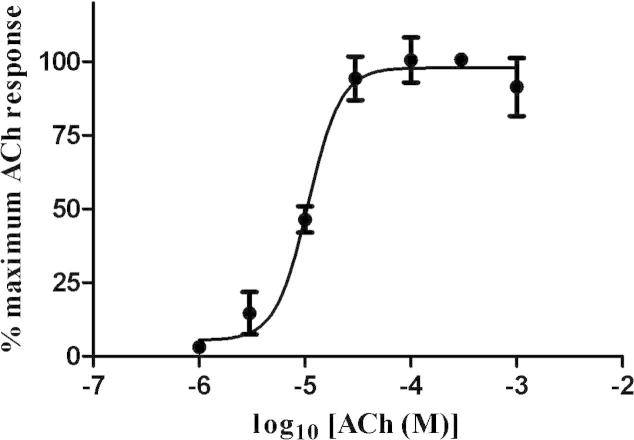
Concentration–response curve for ACh obtained using two-electrode voltage clamp recording from *Xenopus* oocytes expressing the Rsanα1/β2. Data was normalised to the observed peak amplitude of currents recorded in response to 300 μM ACh. Each point represents the mean ± SEM of 6–10 experiments.

**Fig. 7:**
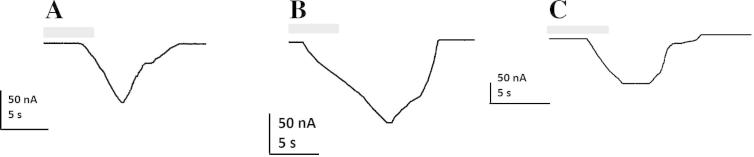
Two-electrode voltage clamp current traces (representative, *n* > 4) from *Xenopus* oocytes expressing Rsanα1/β2 to the potential ligands (A) acetylcholine (ACh, 1 mM), (B) choline (100 μM) and (C) nicotine (100 μM) following a 5 s exposure (grey bar). Traces are obtained from a single oocyte.

Rsanα1 did not form a functional homomeric receptor alone or with the addition of either *C. elegans* or *X. laevis RIC*-3 subunits. However, when co-expressed with the chicken β2 nAChR subunit, a functional heteromeric receptor was produced. The addition of Xla-*RIC*-3 or Cele-*RIC*-3 did not enhance the response in either case. This receptor was not robust and expression was detected in approximately one in 50 oocytes (>1,200 oocytes were tested individually). ACh produced dose-dependent inward currents to Rsanα1/β2 with maximal currents to 1 mM ACh ranging from 20 to 140 nA (Fig. 5A). The EC_50_ of ACh was 10.74 μM (95% Confidence Interval (CI): 8.5–13.5 μM) with a Hill slope of 2.82 (Fig. 6). Rsanα1/β2 was also sensitive to nicotine and choline (Fig. 7). Imidacloprid and spinosad had no effect on Rsanα1/β2 when tested for both agonist (Fig 5B and C) and antagonist activity (not shown).

## 4 Discussion

We report the first known isolated full-length functional nAChR α-subunit from an arachnid. Whilst there is considerable evolutionary distance between insects and the Acari, this nAChR subunit clusters most closely with the α1 insect group (Fig. 2) and contains an insertion in loop F which is typical of the α1 nAChRs (Jones and Sattelle, 2010) and has thus been named Rsanα1.

Breer and Sattelle (1987) found that nAChRs are present at very high concentrations in the insect CNS. Similarly here, however, nested-PCR has shown that Rsanα1 was not solely restricted to the synganglion but was also present in both the Malpighian tubules and oviduct. Despite transcriptomic studies on several tick species, life-stages and tissues (Nene et al., 2004; Anderson et al., 2008; Chmelar et al., 2008; Aljamali et al., 2009; Anatriello et al., 2010; Bissinger et al., 2011; Sonenshine et al., 2011), very few ligand-gated ion channels (LGICs) have been studied. This is not the first report of an arthropod nAChR present outside of the CNS. Using real-time PCR, Gao et al. (2007) found that the presence of *Musca domestica* (Md) Mdα2 nAChR was 150- and 8.5-fold higher in the fly head and thorax than in the abdomen but was nevertheless present at loci other than the head. Notably, Rsanα1 was not detected in the salivary glands which might have been expected as this tissue is highly innervated (Bowman and Sauer, 2004; Simo et al., 2012) and a recent sialotranscriptomic analysis of *Amblyomma maculatum* identified a nAChR β-subunit from this tissue (Karim et al., 2011). However there is no evidence of any effect of nAChR agonists or antagonists on isolated tick salivary glands (Bowman and Sauer, 2004).

It has been proposed that expression of different nAChR subunits varies across development and this has been shown in two α-subunits of *Liposcelis bostrychophila* (Tang et al., 2009). From a control perspective, an ideal drug would be effective against all life stages of tick development. Nested-PCR demonstrated that Rsanα1 is present in all life stages of *R. sanguineus* with the exception of the egg. Roma et al. (2012) have recently shown that the structure of the *R. sanguineus* synganglion is maintained throughout the life-cycle of the tick, from larva to adult. Embryonic nAChR expression has been recorded in insect species including *Locusta migratoria* (Hermsen et al., 1998) and cockroach (*Periplaneta americana*) (Blagburn et al., 1985). In cockroach (Blagburn et al., 1985) and *Drosophila* embryos (Baines and Bate, 1998) nAChRs are expressed prior to synapse formation. It is presumed that Rsanα1 is expressed at a later stage of embryonic development than the eggs used in this study.

Functional expression of arthropod nAChRs is notoriously difficult. Although there are recorded cases of insect α nAChR subunits expressing homomerically in *Xenopus* oocytes (Marshall et al., 1990; Lansdell et al., 2012), most have required the addition of a vertebrate β subunit to permit robust expression (Bertrand et al., 1994; Huang et al., 1999; Liu et al., 2005; Bass et al., 2006; Dederer et al., 2013). This also appears to be necessary for Rsanα1. The low amplitude currents recorded here suggests that there may be other nAChR subunits or co-factors necessary for robust expression. The *RIC-3* gene has been shown to enhance the maturation of some nAChRs from both mammals and invertebrates (Halevi et al., 2002, 2003; Lansdell et al., 2005, 2008, 2012; Bennett et al., 2012) and it has been hypothesised that *X. laevis RIC*-3 may enhance the expression of invertebrate nAChRs within *Xenopus* oocytes (Bennett et al., 2012). However we found that introducing *RIC*-3 made no significant improvement in current amplitudes, suggesting that other currently unknown accessory proteins or subunits are required for more robust expression. The *Xenopus* oocyte system has been proven to be a useful expression system for other members of the arachnid cys-loop LGIC superfamily, having been used to successfully express the *Dermacentor variabilis* GABA receptor RDL (Zheng et al., 2003) and the *Sarcoptes scabiei* pH-gated chloride channel (Mounsey et al., 2007).

We have shown that Rsanα1 is sensitive to ACh, choline and nicotine. A concentration response curve was only obtained for ACh, yielding an EC_50_ of ∼10 μM which is considerably higher than for insects’ α1-nAChRs that have been similarly studied such as *D. melanogaster* (0.07–0.18 μM, Bertrand et al., 1994; Dederer et al., 2011), the cat flea (0.05 μM, Dederer et al., 2011) and the sheep blowfly (0.08 μM, Dederer et al., 2013). It has been previously documented that ticks are sensitive to nicotine as tobacco extracts were historically used as an ectoparasiticide (Meinke, 2001; George et al., 2004) and in the present study we demonstrated that Rsanα1 is responsive to nicotine. Turberg et al. (1996) detected a high-affinity binding site in whole body homogenates of *Rhipicephalus (Boophilus) microplus* larvae for [^3^H] nicotine. These authors also showed that imidacloprid had a very low capacity for displacing [^3^H] nicotine from the *R. microplus* homogenates (Turberg et al., 1996), suggesting that the low binding affinity of imidacloprid in *R. microplus* larval homogenates corresponds to the weak biological efficacy of imidacloprid at the tick nAChR (Lees and Bowman, 2007).

Neonicotinoids also show low toxicity to some spider and mite species including *P. pseudoannulata* (Song et al., 2009) and *Tetranychus urticae* (Dermauw et al., 2012), suggesting that this insensitivity may occur at the nAChR level across the Class Arachnida. Previous studies with insect nAChRs have highlighted that insect β nAChR subunits make an important contribution to neonicotinoid selectivity against insects over vertebrates (Yao et al., 2008). Current studies into this apparent lack of sensitivity in arachnids to imidacloprid have centred on the β-nAChR subunits. In the spider, *P. pseudoannulata,* loop D-F subunit chimeras with *Myzus persicae* and rat β2 nAChRs identified several amino acids which influenced imidacloprid activity but not ACh potency (Song et al., 2009). One key amino acid was glutamine (R81Q) in loop D which caused a significant rightward shift to the imidacloprid concentration–response curve (Song et al., 2009). This glutamine residue is also present in five different tick species, suggesting that this amino acid may play an important role in arachnid insensitivity to imidacloprid (Erdmanis et al., 2012). However, these arachnid studies have focussed solely on the β subunits and not the functional α subunit. We were unable to achieve a highly robust receptor here but the limited functional results obtained indicate that while individual oocytes expressing Rsanα1/ chicken β2 responded to ACh, they showed insensitivity to imidacloprid. In the present study, to ensure that we were only working with oocytes expressing functional Rsanα1, all oocytes were initially tested with ACh. Only those exhibiting responses to ACh were then tested against imidacloprid and other compounds, giving confidence in our interpretation of a lack of response to test compounds.

The spinosyns are effective against tick species and act at a different site from imidacloprid (Salgado, 1998; Nauen et al., 1999). There is growing evidence that they target the arthropod α6 nAChR (Perry et al., 2007; Watson et al., 2010; Hsu et al., 2012; Geng et al., 2013) and it is therefore not surprising that they have no effect on an α1-type receptor.

The first completed arachnid genome has recently been published from the spotted mite *T. urticae* (Grbic et al., 2011). *Tetranychus urticae* contains 10 nAChR subunits consisting of seven α-subunits and three β subunits (Dermauw et al., 2012) but there is no Rsanα1 homologue. However, there is considerable evolutionary distance between *T. urticae* and *R. sanguineus* which are estimated to have diverged 395 million years ago (Hedges et al., 2006; Jeyaprakash and Hoy, 2009). The first completed tick genome from the Prostriate *Ixodes* lineage is soon to be published from *Ixodes scapularis* and it will be interesting to determine the pharmacokinetics of other tick nAChR subunits.

In conclusion, we have isolated and functionally characterised the first known α-nAChR subunit from an arachnid. This subunit, Rsanα1, showed homology to insect α1 nAChRs. It can form a functional nAChR receptor in *Xenopus* oocytes and shows sensitivity to known nAChR ligands but is not a robust receptor. The study of tick LGICs is still at an early stage but ongoing genomic work and future modelling may help to accelerate the development of new and improved acaricides.
